# Compartment-Specific Immune, Endothelial, and Fibrotic Programs in Diabetic Retinopathy: An Integrative Multi-Cohort Transcriptomic Study

**DOI:** 10.3390/biology15181581

**Published:** 2026-09-09

**Authors:** Ece Celik, Buket Yilmaz Bulbul, Burak Andac, Mehmet Celik

**Affiliations:** 1 Division of Allergy and Clinical Immunology, Department of Chest Diseases, Faculty of Medicine, Trakya University, 22030 Edirne, Türkiye; 2 Division of Endocrinology and Metabolism, Department of Internal Medicine, Faculty of Medicine, Trakya University, 22030 Edirne, Türkiye; buketybulbul@trakya.edu.tr (B.Y.B.); burakandac@trakya.edu.tr (B.A.); mehmetcelik@trakya.edu.tr (M.C.)

**Keywords:** diabetic retinopathy, proliferative diabetic retinopathy, single-cell RNA sequencing, endothelial dysfunction, inflammation, extracellular matrix, fibrosis, transcriptomics

## Abstract

Proliferative diabetic retinopathy (PDR) is molecularly heterogeneous, but it is unclear whether immune-rich and endothelial-rich transcriptional patterns recur across retinal compartments. We integrated five public human transcriptomic datasets and analyzed PhysioNet CDED separately as the exploratory clinical context. GSE307925 served as the discovery cohort; GSE245561 and GSE165784 provided independent single-cell cross-cohort evaluation; GSE94019 provided CD31-enriched endothelial evaluation; and GSE160306 provided whole-retina disease-spectrum contextualization. In GSE307925, CD31-low and CD31-high samples separated strongly (PC1 61.5%; exact permutation *p* = 0.0013), with separation retained in all 12 leave-one-out analyses and under TMM normalization. In the two single-cell cohorts, the discovery-derived CD31-low signature localized to myeloid cells and the CD31-high signature to endothelial cells in all nine PDR donors (exact sign-test *p* = 0.00195 for each); the fibrotic/ECM-remodeling core localized to stromal/pericyte cells in all nine donors. In GSE94019, the fibrotic/ECM-remodeling program was increased in PDR (*p* = 0.0028; q = 0.0084) and remained associated with PDR after adjustment for an independent pericyte-lineage score (β = 1.473, 95% CI 0.758–2.189; *p* < 0.001). In GSE160306, the discovery-derived CD31-low program showed a nominal association with advanced DR/DME (β = 0.306, 95% CI 0.026–0.585; *p* = 0.032; q = 0.085). These findings support compartment-dependent molecular enrichment rather than fixed binary patient subtypes.

## Simple Summary

Diabetic retinopathy is often described mainly as a vascular disease, yet advanced proliferative diabetic retinopathy contains immune, vascular, and fibrotic tissue components. We combined five publicly available human transcriptomic datasets to evaluate whether these biological programs recur across distinct retinal compartments. In the discovery cohort, immune-rich and endothelial-rich PDR samples showed a strong transcriptomic separation that was retained after the sequential exclusion of individual samples and under composition-aware normalization. In two independent single-cell cohorts, discovery-derived CD31-low and CD31-high signatures localized consistently to myeloid and endothelial compartments, respectively, across all nine PDR donors, while a fibrotic/ECM-remodeling program localized to stromal/pericyte cells. An independent CD31-enriched cohort showed strong ECM-remodeling in PDR, whereas whole-retina data provided only nominal supportive evidence for an immune-associated increase after correction for multiple testing. Circulating inflammatory and endothelial biomarkers did not reproduce the ocular pattern. These findings support the compartment-dependent enrichment of immune, endothelial, and ECM-remodeling programs rather than fixed patient subtypes.

## 1. Introduction

Diabetic retinopathy (DR) is a major microvascular complication of diabetes and can progress from non-proliferative retinal abnormalities to sight-threatening diabetic macular edema (DME) and proliferative diabetic retinopathy (PDR). PDR is characterized by pathological retinal neovascularization and may be complicated by vitreous hemorrhage, fibrovascular membrane formation, traction, and retinal detachment [1,2,3]. Although vascular endothelial growth factor (VEGF) is central to pathological angiogenesis and anti-VEGF therapy has transformed management, incomplete responses and persistent fibrovascular disease indicate that VEGF signaling alone does not explain the biological complexity of advanced DR [2,3].

The diabetic retina is a multicellular neurovascular tissue in which endothelial cells, pericytes, glial cells, neurons, and infiltrating or resident immune cells interact dynamically [2]. This has shifted the conceptual framework of DR away from an exclusively vascular disorder toward a disease of the retinal neurovascular unit, with inflammation, endothelial dysfunction, metabolic stress, and tissue remodeling contributing in parallel [2,3].

A recent study by McCann et al. provided direct evidence of molecular heterogeneity in end-stage PDR by profiling CD31-positive cells recovered from vitrectomy specimens [4]. Some specimens were enriched for endothelial cells, whereas others were dominated by immune populations. CD31-low samples contained substantial proportions of monocytes, macrophages, T cells, and mast cells, while CD31-high samples expressed markedly higher levels of endothelial markers [4]. The authors proposed that PDR may include biologically distinct forms characterized by the immune-mediated clearance of pathological vessels or persistent endothelial dysfunction, and they further identified mitochondrial dysfunction and ECM organization within the PDR endothelial signature [4].

Single-cell studies of human PDR fibrovascular membranes support the presence of multiple cellular compartments. Corano Scheri et al. identified endothelial, inflammatory, and stromal populations, including angiogenic endothelial cells, proangiogenic macrophages, and a pericyte-to-myofibroblast transition associated with *AEBP1* [5]. Hu et al. reported a myeloid-rich fibrovascular environment and proposed a profibrotic *GPNMB*-positive microglial population [6]; however, a subsequent independent reanalysis questioned the original microglial labeling and emphasized the stromal compartment as the principal source of fibrogenic transcriptional activity [7]. These observations suggest that fibrosis and ECM remodeling warrant explicit consideration alongside immune and endothelial programs.

An additional uncertainty concerns whether signatures obtained from vitreous-associated cells or fibrovascular membranes are detectable in whole retinal tissue. The GSE160306 study profiled human post-mortem retina across the DR spectrum, and showed that molecular changes broaden with disease severity and involve nonvascular retinal cell populations as well as vascular pathways [8]. Consequently, direct replication across compartments should not necessarily require identical global expression patterns; preservation of the expected cell-of-origin or pathway behavior may be more biologically informative.

The present study therefore used a multi-cohort analytical framework to evaluate and extend the recently proposed immune–endothelial model. We reanalyzed GSE307925 to derive treatment-concordant CD31-low and CD31-high transcriptional programs, assessed their cellular localization in two independent single-cell PDR cohorts, evaluated vascular and ECM-remodeling programs in an independent CD31-enriched dataset, and examined disease-stage behavior in whole human retina. A separate PhysioNet cohort was used only as the exploratory clinical context. We hypothesized that immune/myeloid, endothelial/angiogenic, and fibrotic/ECM-remodeling programs would show reproducible but compartment-dependent localization or enrichment across human DR datasets, rather than define fixed patient subtypes.

## 2. Materials and Methods

### 2.1. Study Design and Data Sources

**Figure 1:**
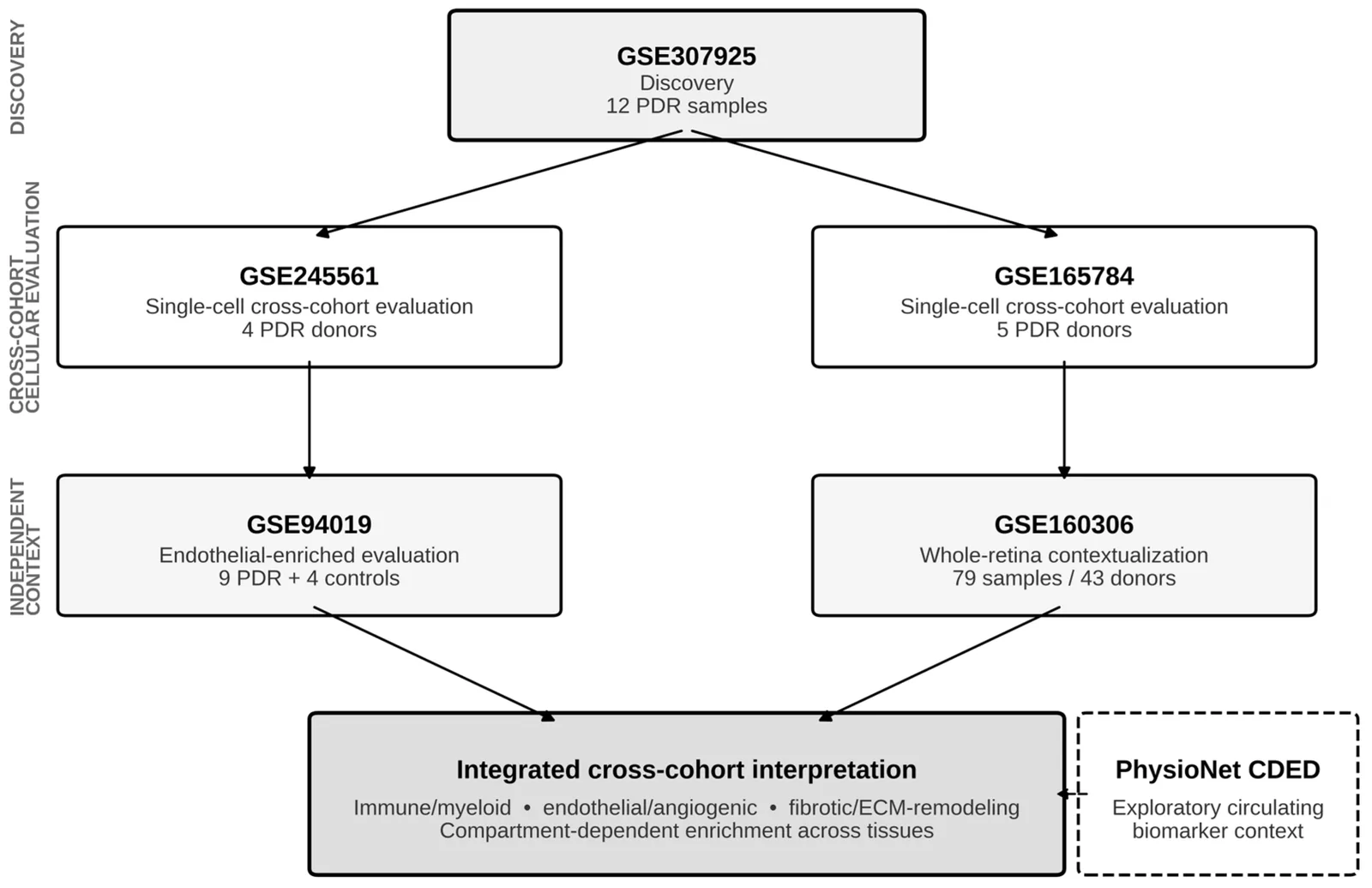
Multi-cohort study architecture. GSE307925 was the discovery cohort; GSE245561 and GSE165784 were independent single-cell cross-cohort evaluation cohorts; GSE94019 provided CD31-enriched endothelial evaluation; GSE160306 provided whole-retina contextualization; and CDED was the exploratory circulating-biomarker context.

This study was an integrative secondary analysis of publicly available, de-identified human datasets. The analytical design was hierarchical: GSE307925 served as the discovery cohort [4]; GSE245561 and GSE165784 were used for independent single-cell cross-cohort evaluation [5,6]; GSE94019 provided a CD31-enriched endothelial evaluation cohort originally generated from CD31-positive cells isolated from PDR fibrovascular membranes and non-diabetic retinal endothelial controls [9]; GSE160306 was used for whole-retina disease-spectrum contextualization [8]; and the PhysioNet Cerebromicrovascular Disease in Elderly with Diabetes (CDED) dataset was used as an exploratory circulating biomarker cohort [10,11,12]. The cohort architecture is shown in Figure 1, and study-level characteristics are summarized in Table 1.

### 2.2. Discovery Cohort: GSE307925

The downloaded GSE307925 count matrix contained 43,836 genomic features across 12 evaluable PDR samples. Five samples were labeled CD31-low (L1–L5) and seven CD31-high (H1–H7) in the source metadata [4]. Preoperative anti-VEGF exposure was also available; three CD31-low and four CD31-high samples had not received preoperative anti-VEGF therapy and were used in a within-cohort treatment-exclusion sensitivity analysis, rather than as an independent validation cohort.

Raw gene counts were normalized for library size and expressed as counts per million (CPM). Genes were retained when CPM was at least 1 in three or more samples, leaving 15,067 genes. For unsupervised principal component analysis (PCA) and signature derivation, expression was transformed as log2(CPM + 0.5). PCA was performed using the 5000 genes with the greatest variance across samples, selected without reference to CD31 group labels.

Transcriptome-wide separation was assessed using the Euclidean distance between group centroids in the first two principal components. Because of the small discovery sample, significance was assessed by exact label permutation while preserving original group sizes. The same procedure was repeated in the anti-VEGF-naive subset, using the same 5000 variable genes selected in the complete discovery cohort. Two reviewer-driven robustness analyses were additionally performed. First, a leave-one-out analysis sequentially removed each of the 12 samples and repeated the PCA, exact permutation procedure, and signature derivation to assess dependence on individual observations. Second, the discovery workflow was repeated after trimmed mean of M-values (TMM) normalization as a composition-aware normalization sensitivity analysis. These analyses were used to characterize robustness and were not treated as an independent validation.

### 2.3. Derivation of Treatment-Concordant Molecular Signatures

To reduce treatment-related confounding, candidate discovery genes were required to show the same direction of CD31-low versus CD31-high effect in both the complete dataset and the anti-VEGF-naive sensitivity subset. Genes were eligible when mean expression was at least 2 CPM and the absolute between-group difference exceeded 1 log2-expression unit in both analyses. Eligible genes were ranked by the smaller of the two effect magnitudes, and the 50 highest-ranking genes in each direction were retained as treatment-concordant CD31-low and CD31-high signatures. Because both comparisons originated from the same discovery cohort, these signatures were treated as candidate molecular programs rather than independent treatment-validated classifiers.

For cross-cohort sensitivity analyses, three compact, pathway-informed canonical cores were defined: an endothelial/angiogenic core (22 genes), an immune/myeloid core (26 genes), and a fibrotic/ECM-remodeling core (17 genes). These sets were designed as representative biological cores rather than comprehensive pathway inventories, diagnostic classifiers, or disease-specific signatures. The endothelial set spans lineage/barrier identity, VEGF/angiogenic signaling, and specialized microvascular programs; the immune/myeloid set spans myeloid lineage, inflammatory/recruitment, and innate effector biology; and the fibrotic/ECM-remodeling set spans structural matrix deposition, matrix organization/turnover, and contractile stromal features. The fibrotic/ECM-remodeling core comprised *COL1A1*, *COL1A2*, *COL3A1*, *COL4A1*, *COL4A2*, *FN1*, *LUM*, *DCN*, *SPARC*, *THBS1*, *COL15A1*, *MMP2*, *SERPINE1*, *CTHRC1*, *AEBP1*, *ACTA2*, and *TAGLN*. Detailed gene set rationale and complete definitions are provided in Supplementary Data S1.

### 2.4. Independent Single-Cell Cross-Cohort Evaluation

GSE245561 contained 9928 cells from four PDR fibrovascular membrane samples [5]. Cells were retained when they expressed 500–5000 genes and had a mitochondrial transcript fraction below 25%, resulting in 6578 QC-retained cells. GSE165784 contained five PDR samples and one proliferative vitreoretinopathy (PVR) sample [6]. The PVR sample was excluded from the primary PDR cross-cohort evaluation; 7525 PDR cells from five donors passed the same quality criteria. The upper detected-gene threshold was retained as a conservative high-complexity multiplet filter; no additional de novo doublet classifier was applied to the downloaded processed matrices.

Broad cellular compartments were assigned using established lineage markers, including endothelial, myeloid/macrophage, T/NK, stromal/pericyte, and B/plasma compartments, with neutrophil-like cells assessed where supported by the dataset. Canonical program scores were calculated from normalized expressions of the represented genes. For each donor, the median score in the biologically expected target compartment was compared with the median score across other major compartments; individual cells were therefore not treated as independent biological replicates. The canonical contrasts were endothelial versus other cells for the endothelial program, myeloid versus other cells for the immune/myeloid program, and stromal/pericyte versus other cells for the fibrotic/ECM-remodeling program. In a reviewer-driven sensitivity analysis, the discovery-derived CD31-low and CD31-high signatures were additionally projected into both single-cell cohorts. To reduce circularity, genes used in broad compartment annotation were removed from the corresponding discovery-signature scores, leaving 45 CD31-low and 35 CD31-high genes. Donor-by-compartment pseudobulk expression was then used to calculate target-versus-other log2-expression contrasts for the CD31-low signature in myeloid cells and the CD31-high signature in endothelial cells. Directional consistency was evaluated at the donor level. Because these single-cell cohorts contained PDR samples only, these analyses assess cellular localization and cross-cohort reproducibility, not disease specificity or association with disease severity.

### 2.5. CD31-Enriched Endothelial Evaluation: GSE94019

GSE94019 contains transcriptomic profiles of CD31-positive cells from nine PDR fibrovascular membranes and four non-diabetic retinal endothelial controls [9]. Both disease and control samples underwent CD31 magnetic-bead enrichment before RNA extraction; they were not cultured endothelial cell preparations. Transcript-level estimates were aggregated to gene symbols where required, normalized for library size to CPM, and transformed as log2(CPM + 0.5). The three compact canonical programs were compared between PDR and control samples. When the fibrotic/ECM-remodeling program showed a significant group-level association, component genes were examined individually. Because CD31 enrichment does not guarantee complete cellular purity, reviewer-driven sensitivity analyses additionally evaluated independent endothelial identity, immune lineage, and pericyte lineage scores. The fibrotic/ECM-remodeling association was then reassessed in a model adjusting for the independent pericyte lineage score to determine whether the signal could be explained solely by residual perivascular/stromal composition.

### 2.6. Whole-Retina Disease-Spectrum Contextualization: GSE160306

The original GSE160306 publication described 80 retinal total-RNA samples from 43 donors [8]. The currently available GEO raw count matrix used here contained 79 samples: 20 controls, 20 diabetes without DR samples, 19 NPDR samples, and 20 advanced NPDR/PDR with DME samples. One NPDR macular sample was absent from the available count matrix. The supplied normalized CPM file contained only 76 samples; therefore, the complete 79-sample raw count matrix was used for all analyses.

Expression values were normalized for library size, converted to CPM, and transformed as log2(CPM + 0.5). For cross-sample module scoring, each component gene was standardized within GSE160306 before calculating the mean program score. Because individual donors could contribute both macular and peripheral retinal samples, associations were evaluated using regression with donor-clustered robust standard errors. The principal model included disease group, retinal site, disease-by-site interaction, age, sex, RNA integrity number (RIN), and post-mortem interval. The prespecified primary contrast compared advanced DR/DME with control tissue in the macula. Disease stage was also examined as an ordinal variable in secondary analysis.

### 2.7. Exploratory Clinical Translation: PhysioNet CDED

The CDED resource is a publicly available multimodal cohort of older adults with and without type 2 diabetes [10,11,12]. At the baseline visit, 44 participants had diabetes; 3 had ungradable retinal assessments, leaving 41 participants for the present retinal analysis (23 without DR and 18 with DR). Circulating biomarkers included soluble intercellular adhesion molecule-1 (sICAM-1), soluble vascular cell adhesion molecule-1 (sVCAM-1), C-reactive protein (CRP), interleukin-6 (IL-6), and tumor necrosis factor-alpha (TNF-α).

Skewed biomarkers were natural-log-transformed and standardized within the diabetic cohort. The systemic endothelial composite was defined as the mean of standardized log sICAM-1 and log sVCAM-1. The systemic inflammatory composite was the mean of standardized log CRP, IL-6, and TNF-α. Composite scores were compared between participants with and without DR and then entered separately into logistic regression models adjusted for HbA1c and diabetes duration. One participant had missing diabetes duration data, yielding n = 40 for adjusted analyses. The CDED files contain a unit-label discrepancy for sICAM-1 between the summary table and data dictionary; because the present analysis used log transformation and within-cohort standardization rather than raw unit interpretation, this discrepancy did not affect relative or z-score-based analyses.

### 2.8. Statistical Analysis

Small independent cohorts were compared using two-sided Mann–Whitney U tests unless otherwise specified. The directional consistency of donor-level single-cell contrasts was evaluated with exact one-sided sign tests. False discovery rate control for related multiple hypotheses used the Benjamini–Hochberg procedure [13]. Hypothesis families were defined by biological role: the three canonical single-cell localization tests formed one family; the two reviewer-driven discovery signature localization tests formed a separate sensitivity family; the three GSE94019 program-level comparisons and the 17 ECM component-gene comparisons were treated as separate families; the five GSE160306 advanced DR/DME program contrasts formed one family; and the two adjusted CDED biomarker-composite models formed an explicitly exploratory family. The GSE160306 ordinal stage trend was treated as a secondary nominal analysis. Effect sizes and uncertainty were emphasized alongside significance testing: discovery separation was summarized with Hedges g; donor-level single-cell results were summarized with median target-versus-other contrasts and bootstrap confidence intervals; GSE94019 was summarized with standardized score differences, bootstrap confidence intervals, and rank-based effect measures; GSE160306 was summarized with regression coefficients and donor-clustered robust 95% confidence intervals; and CDED was summarized with adjusted odds ratios and 95% confidence intervals. Statistical significance was defined as *p* < 0.05, while results that did not remain significant after relevant FDR correction were described as nominal or exploratory rather than confirmatory. Analyses were performed in Python 3.13.5 using NumPy 2.3.5, pandas 2.2.3, SciPy 1.17.0, scikit-learn 1.8.0, statsmodels 0.14.6, and Matplotlib 3.10.8.

### 2.9. Use of Generative Artificial Intelligence

During the preparation and revision of this study, the authors used ChatGPT (OpenAI; GPT-5.6 Sol) to assist with code generation, data organization, statistical analysis implementation, figure preparation, graphical abstract development, and language editing. Generative AI image tools were used to assist in creating the final graphical abstract. All scientific concepts, labels, numerical values, source data, citations, manuscript text, analytical decisions, and final graphical elements were reviewed and verified by the authors, who take full responsibility for the integrity and content of the work.

## 3. Results

### 3.1. GSE307925 Shows a CD31-Low Versus CD31-High Separation Robust to Sample Exclusion and Alternative Normalization

**Figure 2:**
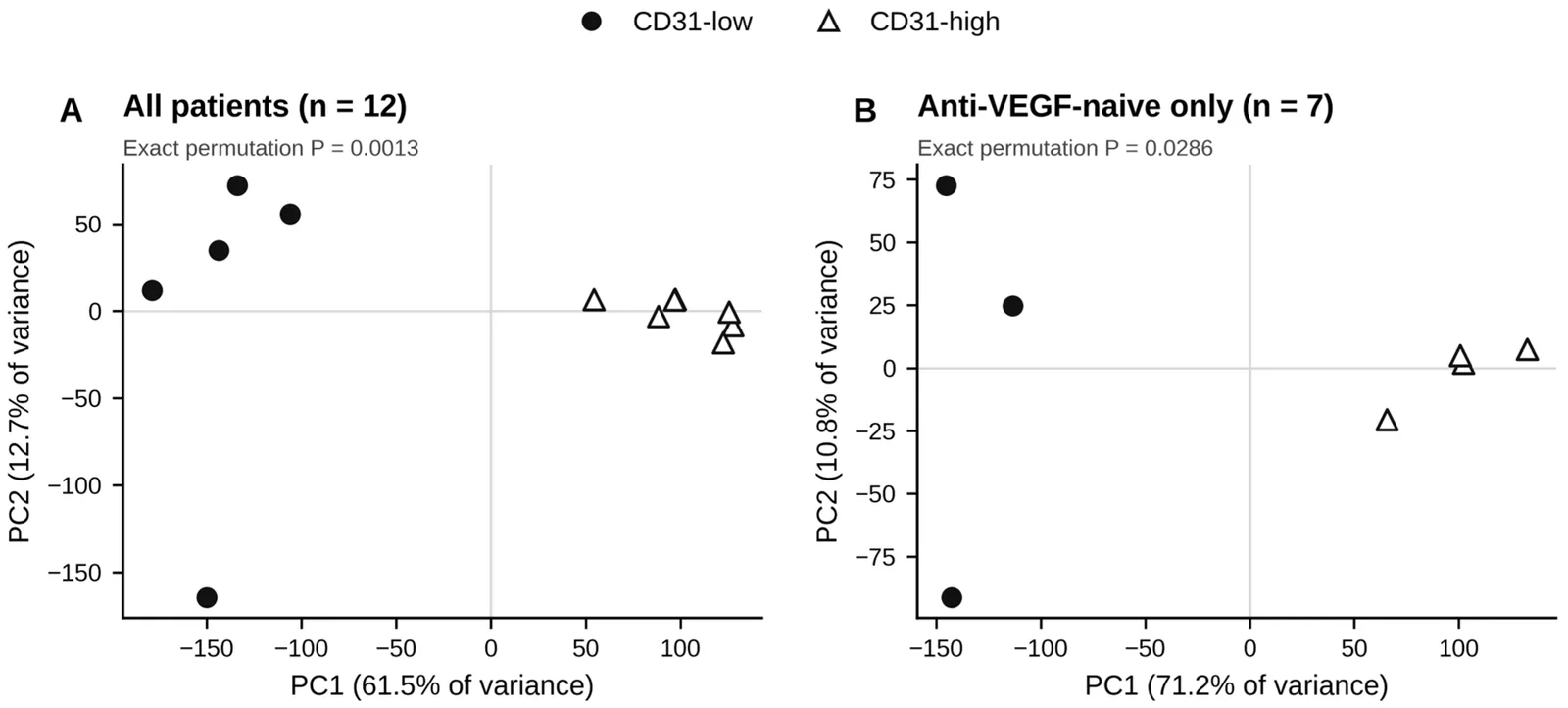
Unsupervised PCA of GSE307925. (**A**) All 12 PDR samples. (**B**) Within-cohort sensitivity analysis restricted to the seven anti-VEGF-naive samples. Filled circles denote CD31-low samples and open triangles denote CD31-high samples. The anti-VEGF-naive analysis evaluates robustness to the exclusion of anti-VEGF-exposed samples and is not an independent validation.

After expression filtering, 15,067 genes were retained in GSE307925. The unsupervised PCA of the 5000 most variable genes showed complete separation of the five CD31-low and seven CD31-high samples along PC1, which explained 61.5% of total variance (Figure 2). Exact multivariate label-permutation analysis confirmed group separation (*p* = 0.00126). The absolute PC1 standardized effect was large (Hedges g = 8.55; bootstrap 95% CI 6.65–17.00), although the confidence interval was wide because of the small discovery sample. In 12 leave-one-out analyses, complete separation was retained after the removal of every individual sample; PC1 explained 59.69–67.65% of variance and exact permutation *p* values ranged from 0.002165 to 0.003030. Under TMM normalization, complete separation was again retained (PC1 = 57.45%; *p* = 0.001263), and 4765/5000 variable genes overlapped the original CPM-based feature set.

The distinction was also evident at canonical endothelial markers. Mean *PECAM1* expression was approximately 11.9-fold higher in CD31-high than CD31-low samples; corresponding fold differences were approximately 576 for *VWF*, 74 for *CDH5*, and 957 for *CLDN5*. Exact Mann–Whitney *p* values were 0.0025 for *PECAM1* and *VWF* and 0.0054 for *CDH5* and *CLDN5*.

Because preoperative anti-VEGF exposure could contribute to transcriptomic variation, PCA and exact permutation analysis were repeated in the untreated subset (three CD31-low versus four CD31-high samples). Separation remained complete; PC1 explained 71.2% of variance, and the exhaustive evaluation of all 35 possible three-versus-four label allocations yielded *p* = 0.02857. Under TMM normalization, PC1 explained 67.93% with the exact same *p* = 0.02857. Signature composition was also highly concordant under TMM (48/50 CD31-low and 50/50 CD31-high genes retained). These analyses support treatment-concordant candidate discovery signatures while avoiding the interpretation of the untreated subset as an independent validation.

### 3.2. Discovery-Derived CD31-Low and CD31-High Signatures Show Reproducible Cellular Localization

**Figure 3:**
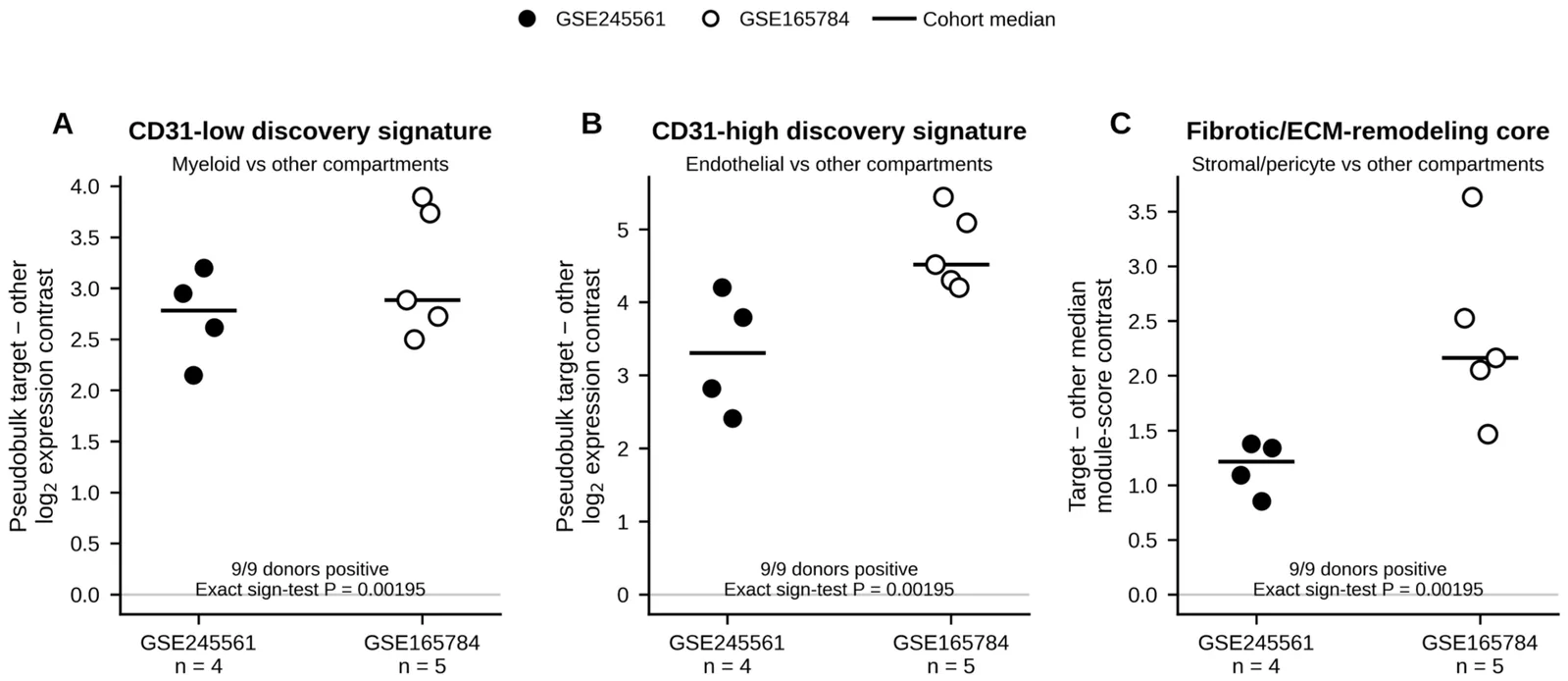
Donor-level single-cell cross-cohort evaluation. (**A**) Discovery-derived CD31-low signature in myeloid versus other compartments. (**B**) Discovery-derived CD31-high signature in endothelial versus other compartments. For panels A and B, annotation marker genes were excluded from the corresponding discovery signature scores and donor-by-compartment pseudobulk contrasts were used. (**C**) Compact fibrotic/ECM-remodeling core in stromal/pericyte versus other compartments. Each point represents one PDR donor; horizontal lines show cohort medians. These analyses assess cellular localization and cross-cohort reproducibility, not PDR specificity or disease severity association.

The discovery-derived signatures were projected into GSE245561 and GSE165784 using donor-by-compartment pseudobulk expression after the removal of broad annotation marker genes from the corresponding signature scores. The CD31-low signature was enriched in the myeloid compartment in all four GSE245561 donors and all five GSE165784 donors. The CD31-high signature was similarly enriched in the endothelial compartment in all nine donors. The compact fibrotic/ECM-remodeling core also localized to stromal/pericyte cells in all nine donors (Figure 3).

Across the two cohorts combined, the discovery-derived CD31-low target-versus-other contrast was positive in nine out of nine donors (median pseudobulk log2-expression contrast 2.885, bootstrap 95% CI 2.501–3.737; exact one-sided sign-test *p* = 0.001953). The discovery-derived CD31-high contrast was also positive in nine out of nine donors (median 4.203, 95% CI 2.820–5.088; *p* = 0.001953). The fibrotic/ECM-remodeling program remained stromal/pericyte-localized in nine out of nine donors (*p* = 0.001953). After BH correction within the respective localization families, these directional results remained FDR-significant. Because the cohorts contain PDR samples only, the findings demonstrate reproducible cellular localization rather than disease specificity.

### 3.3. ECM Remodeling Is Supported in an Independent CD31-Enriched PDR Cohort

**Figure 4:**
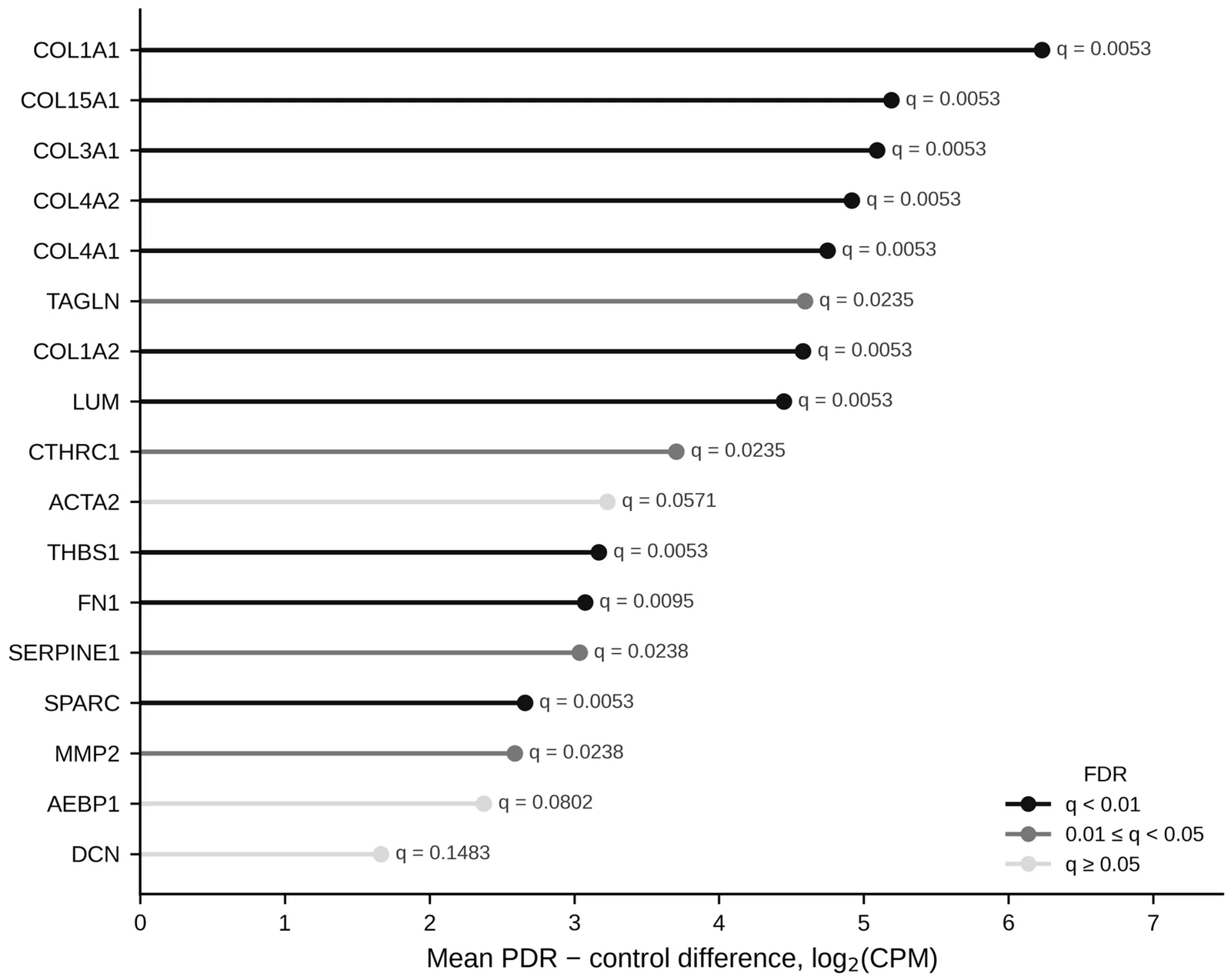
Independent CD31-enriched evaluation of extracellular matrix remodeling in GSE94019. Horizontal lines and points show mean PDR control differences in log2(CPM) for predefined fibrotic/ECM-remodeling genes. Shading indicates the Benjamini–Hochberg-adjusted false discovery rate, and exact q values are annotated.

In GSE94019, the compact fibrotic/ECM-remodeling program was markedly higher in PDR-derived CD31-positive samples than in non-diabetic CD31-enriched retinal controls (Mann–Whitney *p* = 0.002797; Benjamini–Hochberg q = 0.008392 across the three program-level comparisons). All nine PDR samples had a higher fibrotic/ECM-remodeling score than all four controls, corresponding to a rank-biserial correlation of 1.0; the mean standardized score difference was 1.618 (bootstrap 95% CI 1.383–1.863). In contrast, the canonical endothelial and immune/myeloid cores did not differ significantly between groups. Independent lineage score sensitivity analyses showed no reduction in endothelial identity (*p* = 0.825) and there was no significant increase in immune lineage score (*p* = 0.414), whereas an independent pericyte lineage score was higher in PDR (*p* = 0.0196). Importantly, the fibrotic/ECM-remodeling association remained strong after adjustment for the independent pericyte lineage score (adjusted PDR coefficient 1.473, robust 95% CI 0.758–2.189; HC3 *p* = 0.000055). These results indicate that residual perivascular/stromal composition may contribute to, but does not fully account for, the observed ECM-remodeling difference (Figure 4).

The ECM-remodeling association was distributed across multiple transcripts. *COL1A1*, *COL1A2*, *COL3A1*, *COL4A1*, *COL4A2*, *LUM*, *THBS1*, *SPARC*, and *COL15A1* each remained significant after within-module FDR correction (q = 0.0053), and *FN1* remained significant at q = 0.0095. *CTHRC1*, *TAGLN*, *SERPINE1*, and *MMP2* also showed concordant increases with q < 0.025. Thus, the independent signal reflected broad matrix deposition and remodeling rather than a single-gene effect.

### 3.4. The Discovery-Derived CD31-Low Program Shows a Nominal Association with Advanced Whole-Retina Disease

**Figure 5:**
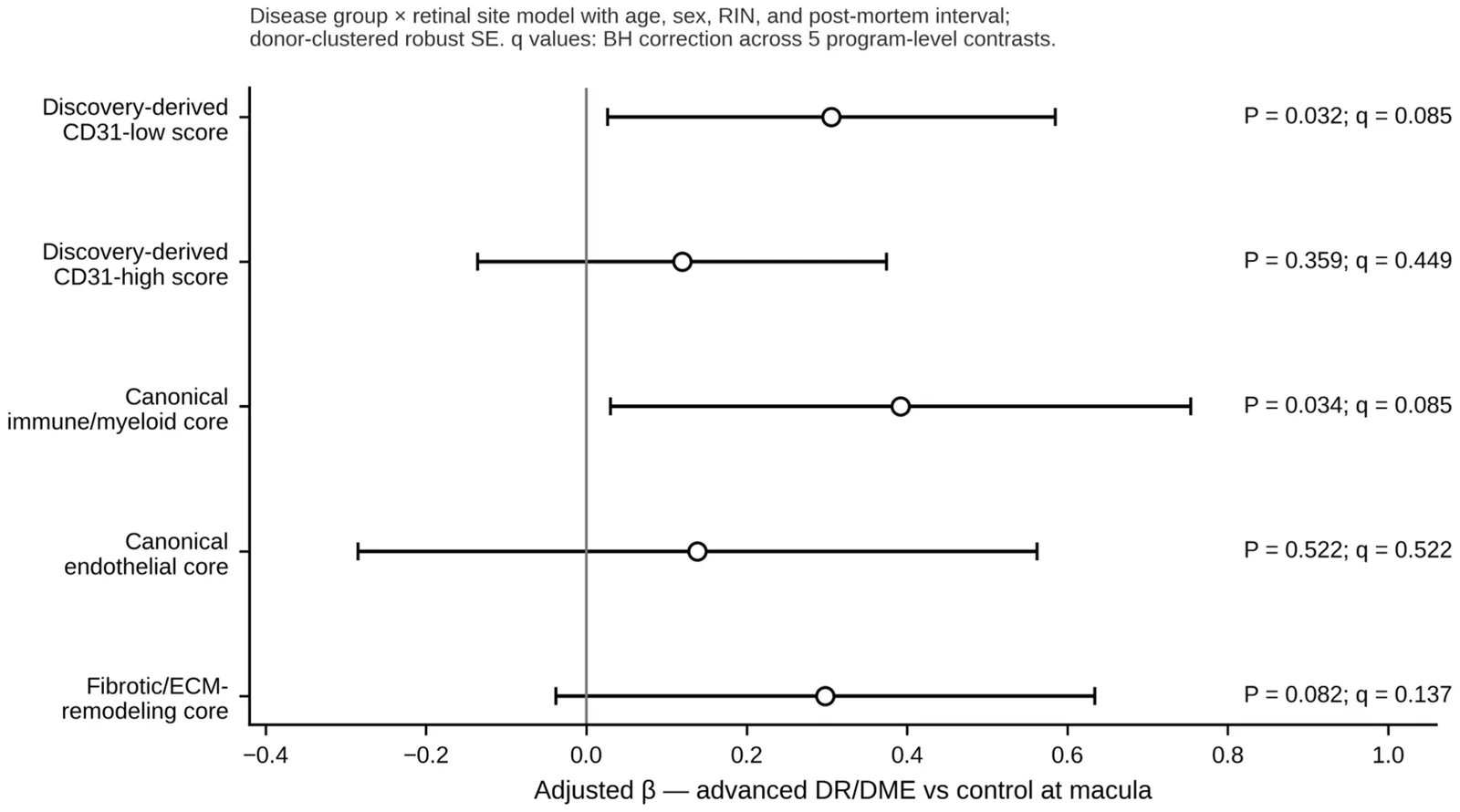
Adjusted associations of predefined molecular programs with advanced DR/DME in GSE160306. Points are regression coefficients and whiskers are 95% confidence intervals. Models included disease group, retinal site, disease-by-site interaction, age, sex, RNA integrity number, and post-mortem interval, with donor-clustered robust standard errors. *p* values are nominal; q values reflect Benjamini–Hochberg correction across the five program-level advanced-disease contrasts.

In GSE160306, regression models accounted for disease group, retinal site, disease-by-site interaction, age, sex, RIN, post-mortem interval, and repeated sampling by donor. The discovery-derived 50-gene CD31-low score was higher in advanced DR/DME than in control macular retina (β = 0.306, 95% CI 0.026–0.585; nominal *p* = 0.0319; Figure 5), and the canonical immune/myeloid core showed a similar nominal association (β = 0.392, 95% CI 0.030–0.754; *p* = 0.034). However, neither association remained significant after BH correction across the five program-level whole-retina contrasts (q = 0.085 for both). These findings are therefore interpreted as supportive disease spectrum contextualization rather than confirmatory cross-tissue replication.

When disease severity was modeled ordinally, the discovery-derived CD31-low score showed a positive nominal stage trend (β = 0.102 per stage; *p* = 0.0445), which was retained as a secondary exploratory analysis. The discovery-derived CD31-high score was not increased in advanced disease (β = 0.119, 95% CI −0.136 to 0.374; *p* = 0.359; q = 0.449), and the canonical endothelial core was also non-significant (*p* = 0.522; q = 0.522). The fibrotic/ECM-remodeling core showed a numerical increase but was not statistically significant (β = 0.298, 95% CI −0.038 to 0.634; *p* = 0.082; q = 0.137).

### 3.5. Circulating Biomarker Composites Do Not Mirror the Ocular Transcriptomic Programs

Among the 41 CDED participants with type 2 diabetes and gradable retinal assessments, 18 had DR and 23 did not. The systemic endothelial composite was numerically lower in participants with DR, but the unadjusted group difference was not significant (*p* = 0.0806). After adjustment for HbA1c and diabetes duration, the OR for DR per 1-SD increase in the endothelial composite was 0.52 (95% CI 0.25–1.06; *p* = 0.0732; q = 0.146 across the two adjusted composite models). This exploratory result was not interpreted as protective.

The systemic inflammatory composite showed no association with DR in unadjusted analysis (*p* = 0.9059) or after adjustment (OR = 1.07, 95% CI 0.53–2.15; *p* = 0.8447; q = 0.8447). Because CDED participants were not the same individuals represented in the transcriptomic cohorts and CDED contains no retinal gene expression measurements, these results were interpreted in the exploratory cross-compartment context rather than molecular validation (Supplementary Data S1, worksheet ‘CDED_Exploratory’).

Baseline clinical and laboratory characteristics according to diabetic retinopathy status are summarized in Table 2. No individual characteristic differed significantly between groups at the *p* < 0.05 level, although platelet count and previous tobacco use showed nonsignificant numerical differences.

### 3.6. Integrated Cross-Cohort Synthesis

The cross-cohort pattern was not consistent with a single uniformly expressed inflammatory or vascular state. Instead, discovery-derived CD31-low and CD31-high programs showed reproducible localization to myeloid and endothelial compartments across independent single-cell datasets, respectively; the whole-retina CD31-low association was supportive but only nominal after multiplicity correction; and fibrotic/ECM-remodeling was most strongly supported in stromal/pericyte and CD31-enriched pathological vascular contexts (Table 3).

## 4. Discussion

This integrative multi-cohort analysis extends the recently proposed immune endothelial model of PDR by evaluating its reproducibility across anatomically and technically distinct human datasets. Three main observations emerged. First, discovery-derived CD31-low and CD31-high programs mapped reproducibly to myeloid and endothelial compartments in two independent single-cell cohorts, even after annotation-marker genes were excluded from the corresponding discovery signature scores. Second, fibrotic/ECM-remodeling emerged as an additional and particularly reproducible component of advanced PDR biology, localizing to stromal/pericyte populations and showing strong independent enrichment in CD31-enriched PDR samples. Third, these programs were not expressed uniformly across compartments: the immune-associated whole-retina signal was only nominal after multiple-testing correction, whereas endothelial and ECM-remodeling signals were more anatomically restricted.

### 4.1. Reframing the Immune Endothelial Dichotomy as a Compartment-Dependent Architecture

McCann et al. proposed that end-stage PDR is heterogeneous, with some CD31-positive specimens dominated by endothelial cells and others by immune populations [4]. Our results support the biological basis of this observation: across nine independent PDR donors from two single-cell datasets, the discovery-derived CD31-high signature consistently localized to endothelial cells and the CD31-low signature to the myeloid compartment. This cross-cohort localization remained evident after annotation-marker genes were removed from the corresponding discovery signature scores, reducing the concern that the finding simply reflected canonical lineage markers.

However, our data do not support treating these signals as fixed binary patient subtypes or as evidence of direct molecular interaction among programs. Whole-retina tissue did not reproduce a simple reciprocal immune-versus-endothelial pattern, and the source study itself did not identify readily distinguishable clinical characteristics between CD31-high and CD31-low patients [4]. A more conservative interpretation is therefore that these molecular programs show compartment-dependent localization or enrichment whose relative magnitude varies with cellular composition, anatomical compartment, disease stage, and possibly treatment exposure.

### 4.2. ECM Remodeling Emerges as an Additional Reproducible Program

The most important extension beyond the binary model was the independent fibrotic/ECM-remodeling signal. In both single-cell cohorts, the compact ECM-remodeling core localized most strongly to stromal/pericyte populations in every donor. In GSE94019, the same compact program was markedly higher in PDR-derived CD31-positive samples than in non-diabetic CD31-enriched retinal controls, with all nine PDR samples exceeding all four controls. The label “fibrotic/ECM-remodeling” is used deliberately: the gene set combines structural collagens and matrix organizers with genes involved in matrix turnover and contractile stromal activation, and is not intended to imply that every component is fibrosis-specific.

This finding is consistent with the original McCann PDR endothelial signature, which included enrichment of ECM organization and collagen–fibril assembly pathways [4]. It is also concordant with independent single-cell work identifying pericyte-to-myofibroblast transition and *AEBP1*-associated fibrogenesis in human PDR fibrovascular membranes [5]. A subsequent reanalysis of GSE165784 likewise argued that stromal cells, rather than a uniquely profibrotic microglial subset, are the dominant fibrogenic compartment [7]. Our donor-level results align with this more conservative interpretation while still supporting a substantial myeloid inflammatory component.

The GSE94019 result is compatible with evidence of endothelial-to-mesenchymal transition in PDR, but the reviewer-driven cellular-composition analysis also indicates that residual perivascular/stromal contribution cannot be excluded. The independent endothelial identity score was preserved, while the pericyte lineage score was higher in PDR. Importantly, the ECM-remodeling association remained strong after adjustment for the independent pericyte lineage score. We therefore interpret the signal as compatible with contributions from both pathological endothelial state and residual stromal/perivascular composition, rather than attributing it exclusively to endothelial cells. This interpretation is also consistent with that of Zou et al., who reported increased mesenchymal markers, including *COL1A2*, together with reduced endothelial *CDH5* and increased inflammatory/autophagy-related proteins in PDR [14].

### 4.3. The Immune Program Extends into Whole-Retina Advanced Disease

The behavior of the immune program differed from that of the endothelial and ECM-remodeling programs. In GSE160306, the discovery-derived CD31-low signature and the canonical immune/myeloid core each showed nominal associations with advanced DR/DME after adjustment for retinal region, age, sex, RIN, post-mortem interval, and donor clustering. However, neither remained significant after BH correction across the five whole-retina program-level contrasts (q = 0.085 for both). These results therefore provide a supportive disease-spectrum context rather than confirmatory evidence. The direction is nevertheless consistent with the original GSE160306 study, which found the largest transcriptional perturbations in later-stage DR and emphasized biological changes extending beyond a purely vascular model [8].

By contrast, neither the discovery-derived CD31-high program nor the canonical endothelial core showed a comparable whole-retina increase, and the fibrotic/ECM-remodeling program showed only a non-significant numerical trend. These differences are biologically plausible because endothelial and stromal cells constitute relatively restricted populations within the whole retina, so signals from pathological vascular or fibrovascular compartments may be diluted by neuronal, glial, and other retinal transcripts. Cross-compartment evaluation is therefore better interpreted as the preservation of expected cellular localization and pathway behavior than as a requirement for identical whole-tissue effect sizes.

### 4.4. Mitochondrial Dysfunction May Be More Context Dependent than ECM Remodeling

The source study identified mitochondrial dysfunction, suppressed oxidative phosphorylation, and impaired respiratory electron transport as prominent features of PDR endothelium [4]. In our independent GSE94019 analyses, however, ECM remodeling was substantially more reproducible than a generalized endothelial identity shift, and exploratory mitochondrial/OXPHOS scoring did not provide convincing independent replication. This does not disprove the role of mitochondrial dysfunction; rather, it suggests that metabolic abnormalities may be more sensitive to disease stage, cell state, sampling compartment, or treatment context than the ECM program. Notably, McCann et al. also found that the mitochondrial component of the human PDR signature was not reproduced in the murine oxygen-induced retinopathy model [4], highlighting the broader problem of context dependence in translational retinal biology.

### 4.5. Ocular Programs Are Not Simply Mirrored in Peripheral Blood

The exploratory CDED analysis showed no corresponding increase in circulating inflammatory or endothelial composites among participants with DR. This should not be considered a failed replication because the biological compartments, measurement platforms, and individuals were entirely different. Retinal transcriptomics reflects local cellular states, whereas circulating CRP, IL-6, TNF-α, sICAM-1, and sVCAM-1 integrate systemic processes. The absence of a parallel blood signal therefore encourages caution against assuming that peripheral biomarkers directly represent the molecular complexity of local retinal pathology. Larger cohorts with matched ocular and systemic measurements are required to test this relationship directly.

### 4.6. Strengths and Limitations

Several features strengthen this study. The analysis was organized around biologically defined roles for each dataset rather than pooling heterogeneous cohorts indiscriminately. Discovery was separated from independent single-cell cross-cohort evaluation, CD31-enriched endothelial evaluation, whole-retina contextualization, and exploratory clinical translation. Two independent single-cell cohorts were used, and the donor rather than the individual cell was retained as the inferential unit, minimizing pseudoreplication. Reviewer-driven analyses further evaluated dependence on individual discovery samples, sensitivity to TMM normalization, circularity in single-cell signature localization, residual cellular composition in GSE94019, multiple-testing burden, and effect-size uncertainty. Concordant and discordant findings were retained rather than forcing every dataset to support the same pathway.

The study also has important limitations. All analyses are retrospective secondary analyses of public datasets generated using different tissue sources, library preparation methods, sequencing platforms, patient populations, disease stages, and treatment exposures; causal relationships cannot be inferred. The GSE307925 discovery cohort contains only 12 samples, and the seven anti-VEGF-naive samples are a subset of the same cohort rather than an independent validation set. Although the leave-one-out and TMM sensitivity analyses indicate that the global separation is not driven by a single sample or the original normalization strategy, the exact gene ranking—particularly for the CD31-low signature—remains subject to small-sample uncertainty. The two single-cell cohorts include only nine independent PDR donors and lack non-PDR controls or longitudinal disease stages, so they support cellular localization but not PDR specificity or severity association. GSE94019 includes only four non-diabetic controls, and CD31 enrichment does not guarantee complete purity; the higher pericyte lineage score indicates that residual stromal/perivascular composition may contribute to the ECM-remodeling signal, although adjustment for this score did not eliminate the association. The whole-retina CD31-low and immune associations did not remain significant after multiple-testing correction, and are therefore considered nominal contextual findings. Finally, CDED participants were unrelated to the transcriptomic cohorts, and the fibrotic/ECM-remodeling program should be interpreted as a reproducible remodeling axis rather than proof of a distinct clinical fibrotic subtype or causal mechanism.

Future work should prospectively evaluate these programs in larger cohorts with matched retinal imaging, vitreous or membrane sampling, systemic biomarkers, and treatment-response data. Additional independent PDR single-cell and CD31-enriched endothelial datasets would be particularly valuable when they become available. Spatial transcriptomics and single-nucleus approaches may help determine whether immune, endothelial, and ECM-remodeling programs coexist within the same lesions, or occupy distinct microanatomical niches.

## 5. Conclusions

Across independent human bulk and single-cell datasets, discovery-derived immune/myeloid and endothelial transcriptional programs show reproducible cell-of-origin localization in PDR, while an additional fibrotic/ECM-remodeling program is consistently observed within stromal/pericyte populations and CD31-enriched pathological vascular tissue. Whole-retina data provide only nominal supportive evidence for an immune-associated increase after multiple-testing correction, whereas endothelial and ECM-remodeling signals are more anatomically restricted. These findings support the compartment-dependent localization and enrichment of molecular programs in advanced diabetic retinopathy rather than fixed binary patient subtypes, and identify ECM remodeling as a particularly reproducible component of the PDR molecular landscape.

## Figures and Tables

**Table 1:** Public datasets included in the integrative analysis.

Dataset	Material/Data Type	Analyzed Sample Size	Role	Primary Endpoint
GSE307925	Bulk RNA-seq of vitreous-derived CD31+ cells	12 PDR samples (5 CD31-low; 7 CD31-high)	Discovery	Treatment-concordant CD31-low and CD31-high programs
GSE245561	scRNA-seq of PDR fibrovascular membranes	4 PDR donors; 6578 QC-retained cells	Single-cell evaluation 1	Cellular localization of canonical and discovery-derived programs
GSE165784	scRNA-seq of proliferative retinal membranes	5 PDR donors; 7525 QC-retained PDR cells	Single-cell evaluation 2	Independent donor-level localization
GSE94019	Endothelial-enriched RNA-seq	9 PDR + 4 non-diabetic controls	CD31-enriched endothelial evaluation	ECM-remodeling and cellular-composition sensitivity
GSE160306	Bulk total-RNA from human retina	79 samples from 43 donors	Disease-spectrum contextualization	Program behavior across DR severity
CDED	Clinical/serum biomarkers and retinal grading	41 gradable participants with type 2 diabetes	Exploratory translation	Circulating endothelial/inflammatory composites vs. DR

**Table 2:** Clinical and laboratory characteristics of the PhysioNet CDED cohort according to diabetic retinopathy status.

Variable	No DR (n = 23)	DR (n = 18)	*p* Value
**Clinical characteristics**			
Diabetes duration, years	10 (8–16) [n = 23]	11 (7–15) [n = 17]	0.902
BMI, kg/m^2^	28.35 (26.57–32.50)	30.41 (27.07–34.46)	0.379
Hypertension	17/22 (77.3%)	13/17 (76.5%)	1.000
Previous tobacco use	10/18 (55.6%)	4/16 (25.0%)	0.092
Current tobacco use	1/23 (4.3%)	3/18 (16.7%)	0.303
Insulin treatment	5/23 (21.7%)	5/18 (27.8%)	0.725
**Metabolic, hematologic, and renal variables**			
Glucose, mg/dL	118.5 (99.8–146.5) [n = 22]	112.0 (92.0–126.0) [n = 17]	0.453
HOMA1-IR	3.20 (2.49–4.78) [n = 22]	2.86 (1.36–3.83) [n = 17]	0.229
HbA1c, %	6.70 (6.30–7.75)	7.30 (6.53–8.03)	0.221
WBC, K/uL	5.70 (4.60–7.50)	6.70 (5.23–7.08)	0.365
Platelets, K/uL	215 (189–250.5)	257.5 (215.3–282.3)	0.076
Urine albumin/creatinine ratio, mg/g	6.10 (3.75–12.85)	7.40 (3.35–10.58)	0.763
**Endothelial and inflammatory biomarkers**			
sICAM-1, source-reported units	216.0 (177.7–236.0)	190.9 (179.4–211.0)	0.118
sVCAM-1, ng/mL	728.58 (629.88–939.72)	623.52 (570.78–785.99)	0.118
CRP, mg/L	0.99 (0.55–1.94)	1.03 (0.30–3.41)	0.979
IL-6, pg/mL	2.75 (2.23–3.10)	2.70 (2.00–4.02)	0.572
TNF-alpha, pg/mL	1.40 (1.00–1.65)	1.20 (1.02–1.48)	0.653

Data are median (interquartile range) unless otherwise indicated; categorical variables are n/N (%). *p*-values were calculated using two-sided Mann–Whitney U tests for continuous variables and Fisher exact tests for categorical variables. Denominators vary because of missing data. The CDED source files contain a unit-label discrepancy for sICAM-1; therefore, sICAM-1 is reported here in source-reported units. DR, diabetic retinopathy; BMI, body mass index; HOMA1-IR, homeostatic model assessment of insulin resistance; WBC, white blood cell count; sICAM-1, soluble intercellular adhesion molecule-1; sVCAM-1, soluble vascular cell adhesion molecule-1; CRP, C-reactive protein; IL-6, interleukin-6; TNF-alpha, tumor necrosis factor-alpha.

**Table 3:** Cross-cohort synthesis of the three molecular programs.

Program	Discovery	Independent scRNA Evaluation	Independent Tissue Evaluation	Integrated Interpretation
Immune/myeloid	CD31-low enriched	CD31-low signature → myeloid in 9/9 donors	Whole-retina CD31-low/immune: nominal only (q = 0.085)	Reproducible localization; supportive whole-retina context
Endothelial/angiogenic	CD31-high enriched	CD31-high signature → endothelial in 9/9 donors	No whole-retina increase; endothelial identity preserved in GSE94019	Strong compartment identity; disease signal is context-dependent
Fibrotic/ECM-remodeling	Present within PDR endothelial signature	ECM-remodeling core → stromal/pericyte in 9/9 donors	GSE94019 increase (q = 0.0084); persists after pericyte-lineage adjustment	Reproducible remodeling; endothelial and perivascular contributions are possible

## Data Availability

All transcriptomic datasets analyzed in this study are publicly available through the NCBI Gene Expression Omnibus under accession numbers GSE307925, GSE245561, GSE165784, GSE94019, and GSE160306. The exploratory clinical dataset is publicly available from PhysioNet as Cerebromicrovascular Disease in Elderly with Diabetes (CDED), version 1.0.1, https://doi.org/10.13026/00bm-0x81. No new primary participant-level data were generated. To avoid the unnecessary redistribution of third-party source datasets, the public code repository will not contain copies of the GEO or PhysioNet data. Instead, it will provide the complete analysis scripts, environment specifications, gene set definitions, and figure-generation code, together with instructions and official accession numbers/DOIs required to obtain the source data directly from their original repositories. Repository URL: https://github.com/ececelik39/diabetic-retinopathy-multicohort-transcriptomics (accessed on 6 September 2026).

## Supplementary Materials

The following supporting information can be downloaded at: https://www.mdpi.com/article/10.3390/biology15181581/s1, Data S1, containing cohort definitions, discovery and normalization sensitivity analyses, donor-level single-cell analyses, GSE94019 cellular composition sensitivity analyses, effect sizes and multiplicity control, gene set rationale, and cross-cohort synthesis.

## Abbreviations

The following abbreviations are used in this manuscript:

CDED	Cerebromicrovascular Disease in Elderly with Diabetes
CPM	counts per million
CRP	C-reactive protein
DME	diabetic macular edema
DR	diabetic retinopathy
ECM	extracellular matrix
FDR	false discovery rate
FVM	fibrovascular membrane
NPDR	non-proliferative diabetic retinopathy
OR	odds ratio
PCA	principal component analysis
PDR	proliferative diabetic retinopathy
PVR	proliferative vitreoretinopathy
RIN	RNA integrity number
scRNA-seq	single-cell RNA sequencing
sICAM-1	soluble intercellular adhesion molecule-1
sVCAM-1	soluble vascular cell adhesion molecule-1
TNF-α	tumor necrosis factor-alpha
VEGF	vascular endothelial growth factor
